# Targeting the Biology of Aging in Cerebrovascular Disease: Inflammation, Metabolism, Senescence, and Regeneration

**DOI:** 10.3390/ijms27041880

**Published:** 2026-02-15

**Authors:** Daniela Glavan, Thorsten R. Doeppner, Mihaela Abuzan, Dirk M. Hermann, Bogdan Capitanescu, Denisa Greta Olaru, Aurel Popa-Wagner

**Affiliations:** 1 Department of Neurology, University Medical Center Göttingen, 37075 Göttingen, Germany; danielaglavan@umfcv.ro (D.G.); thorsten.doeppner@neuro.med.uni-giessen.de (T.R.D.); 2 Clinic of Psychiatry, University of Medicine and Pharmacy of Craiova, 200349 Craiova, Romania; 3 Department of Neurology, University of Giessen Medical School, 35392 Giessen, Germany; 4 Experimental Research Center for Normal and Pathological Aging (ARES), University of Medicine and Pharmacy of Craiova, 200349 Craiova, Romania; mihaelaabuzan@yahoo.com (M.A.); dirk.hermann@uk-essen.de (D.M.H.); bogdanc26@yahoo.com (B.C.); 5 Chair of Vascular Neurology and Dementia, Department of Neurology, University Hospital Essen, 45147 Essen, Germany

**Keywords:** ageing, cerebrovascular diseases, inflammation, metabolism, regeneration

## Abstract

Aging is the strongest independent risk factor for cerebrovascular diseases, profoundly influencing vascular structure, immune responses, and regenerative capacity of the brain. Traditional therapeutic strategies, largely developed in younger populations, often show reduced efficacy and increased risk in elderly patients, underscoring the need for age-adapted interventions. Advances in the understanding of cerebrovascular aging have revealed key mechanisms such as vascular senescence, chronic low-grade inflammation, blood–brain barrier dysfunction, mitochondrial impairment, and circadian dysregulation as central drivers of disease progression and poor recovery. This narrative review summarizes emerging therapeutic strategies targeting the molecular and cellular hallmarks of aging-related cerebrovascular disease. These include immunomodulatory and anti-inflammatory approaches, senescence-targeted therapies, stem cell and extracellular vesicle-based regenerative strategies, RNA-based interventions, and metabolic and mitochondrial modulation. Particular emphasis is placed on therapies aimed at restoring neurovascular unit integrity and promoting brain repair in the aged microenvironment. Additionally, this review highlights the growing role of chronobiology and precision medicine, integrating biomarkers and multi-omics approaches to tailor treatments for elderly patients. Collectively, these emerging therapies represent a paradigm shift from symptom-oriented management toward mechanism-based and personalized interventions. Addressing age-specific pathophysiology will be critical for improving outcomes in cerebrovascular diseases in the aging population and for translating experimental advances into effective clinical therapies.

## 1. Introduction

Aging is the most powerful non-modifiable risk factor for cerebrovascular diseases, including ischemic stroke, cerebral small vessel disease, and vascular cognitive impairment. The progressive increase in life expectancy worldwide has led to a disproportionate rise in cerebrovascular morbidity and mortality among older adults, making age-related vascular brain injury a major public health challenge. Importantly, aging not only increases disease incidence but fundamentally alters cerebrovascular structure, immune responses, metabolic resilience, and regenerative capacity, thereby shaping disease severity and recovery trajectories.

At the molecular level, cerebrovascular aging reflects the cumulative impact of interconnected biological hallmarks of aging, including genomic instability, mitochondrial dysfunction, cellular senescence, epigenetic alterations, and chronic low-grade inflammation [1]. Within the cerebral vasculature, these processes manifest as endothelial dysfunction, impaired nitric oxide signaling, increased oxidative stress, and progressive breakdown of the blood–brain barrier [BBB]. Such changes compromise cerebral autoregulation and neurovascular coupling, rendering the aging brain particularly vulnerable to ischemic and hypoxic insults [2]. A central feature of aging-related cerebrovascular disease is the dysregulation of the neurovascular unit (NVU), defined as a dynamic and functionally integrated ensemble composed of endothelial cells, pericytes, astrocytes, neurons, microglia, and extracellular matrix components that collectively regulate cerebral blood flow, metabolic coupling, immune surveillance, and blood–brain barrier integrity. Aging disrupts bidirectional signaling within this unit, leading to impaired metabolic support, altered immune surveillance, and reduced adaptive plasticity [3]. Concomitantly, the aging immune system undergoes profound remodeling characterized by immunosenescence and inflammaging, marked by persistent activation of innate immune pathways and exaggerated microglial responses following injury [4]. This chronic inflammatory milieu exacerbates secondary brain injury after stroke and limits functional recovery.

Despite major advances in acute cerebrovascular care, current therapeutic strategies remain suboptimal for elderly patients. Reperfusion therapies, including intravenous thrombolysis and mechanical thrombectomy, show reduced efficacy and increased complication rates with advancing age, in part due to vascular fragility, comorbidities, and delayed treatment presentation [5]. Moreover, most neuroprotective and regenerative strategies have failed to translate clinically, partly because preclinical studies often rely on young animal models that do not recapitulate the aging cerebrovascular environment.

Emerging insights from molecular gerontology, neuroimmunology, and regenerative medicine have identified aging-specific therapeutic targets that extend beyond traditional vascular risk factor control. These include interventions aimed at modulating chronic neuroinflammation, eliminating or reprogramming senescent cells, restoring mitochondrial and metabolic function, enhancing endogenous repair via stem cell-derived paracrine signaling, and leveraging extracellular vesicles and RNA-based communication systems. Additionally, growing evidence implicates circadian rhythm disruption as a contributor to cerebrovascular vulnerability in aging, opening new avenues for chronobiology-informed therapies [6].

This narrative review synthesizes current knowledge on emerging treatments for aging-related cerebrovascular diseases, with a particular emphasis on molecular mechanisms, experimental evidence, and translational challenges. By integrating advances across vascular biology, aging research, and neuroregeneration, this review aims to provide a framework for developing age-adapted, mechanism-driven therapeutic strategies to improve outcomes in the growing elderly population.

## 2. Methods

This narrative review was conducted following established methodological recommendations for non-systematic and mechanistic reviews in biomedical research, with the aim of synthesizing emerging molecular and cellular therapeutic strategies targeting aging-related cerebrovascular diseases. Given the integrative and hypothesis-generating nature of this work, a qualitative, thematic approach was employed rather than a formal systematic review or meta-analysis [7].

### 2.1. Literature Search Strategy

A comprehensive literature search was performed using PubMed/MEDLINE, Web of Science, and Scopus databases. Searches were conducted between January 2024 and September 2025 and included peer-reviewed articles published primarily from 2015 onward, with emphasis on recent advances (2020–2025). Search terms were combined using Boolean operators and included both Medical Subject Headings [MeSH] and free-text keywords related to aging, cerebrovascular disease, and emerging therapies. Core search terms included: aging, cerebrovascular aging, ischemic stroke, small vessel disease, neurovascular unit, blood–brain barrier, inflammaging, immunosenescence, cellular senescence, senolytics, stem cells, extracellular vesicles, microRNA, mitochondrial dysfunction, metabolism, circadian rhythm, and chronotherapy.

The search strategy was designed to capture studies addressing aging-specific molecular mechanisms, experimental interventions in aged animal models, and translational or early clinical evidence relevant to elderly populations.

### 2.2. Eligibility Criteria

Studies were selected for inclusion based on their relevance to aging-related cerebrovascular diseases and their contribution to understanding molecular, cellular, and translational mechanisms underlying disease pathophysiology and therapeutic intervention. Eligible publications were restricted to peer-reviewed articles written in English and included original research studies, systematic reviews, and high-quality narrative reviews. Particular emphasis was placed on investigations addressing age-dependent alterations in cerebrovascular structure and function, neurovascular unit integrity, immune regulation, metabolic homeostasis, and regenerative capacity, in line with contemporary concepts of cerebrovascular aging [3]. Priority was given to experimental studies employing aged animal models or in vitro systems designed to recapitulate aging-associated phenotypes, as well as to clinical and translational studies involving cohorts enriched for older adults. This approach was adopted to mitigate a well-recognized limitation in cerebrovascular research, namely the frequent reliance on young experimental models that fail to capture aging-specific disease mechanisms [8]. Studies focusing exclusively on young or developmentally immature models were included only when their mechanistic insights were directly applicable to aging-related cerebrovascular processes. Conference abstracts, editorials, commentaries, and non-peer-reviewed sources were excluded. When overlapping or conflicting findings were identified, greater weight was assigned to studies with robust experimental design, molecular validation, and higher translational relevance to aging populations [9].

### 2.3. Data Extraction and Thematic Synthesis

Relevant information was extracted manually from all eligible studies and synthesized using a qualitative, thematic approach. Data extraction focused on molecular pathways, cellular mechanisms, and therapeutic targets implicated in aging-related cerebrovascular dysfunction, with particular attention to processes known to be fundamentally altered with aging, including chronic low-grade inflammation, immunosenescence, cellular senescence, mitochondrial dysfunction, and metabolic dysregulation [1]. In addition, emphasis was placed on mechanisms governing neurovascular unit communication, blood–brain barrier integrity, and post-injury repair responses, which are increasingly recognized as critical determinants of outcome in elderly patients [2]. Rather than quantitatively aggregating experimental outcomes, the extracted data were integrated to construct a coherent mechanistic framework linking biological aging to cerebrovascular disease progression and therapeutic responsiveness. Findings were interpreted within established models of vascular aging and neuroinflammation, allowing for critical comparison across experimental paradigms and disease contexts [4]. This integrative synthesis was designed to identify convergent molecular pathways, highlight emerging therapeutic strategies, and delineate key translational challenges that must be addressed to effectively bridge experimental discoveries and clinical application in aging populations.

## 3. Pathophysiology of Aging-Related Cerebrovascular Diseases

Neuroinflammation represents a central and sustained driver of aging-related cerebrovascular pathology. Aging is accompanied by immunosenescence and chronic low-grade inflammation (*inflammaging*), characterized by persistent activation of innate immune pathways and exaggerated inflammatory responses to injury [10]. Experimental studies have demonstrated that microglia in the aged brain adopt a primed phenotype, exhibiting heightened sensitivity to ischemic and vascular insults and producing excessive levels of pro-inflammatory cytokines, reactive oxygen species, and complement components [10,11,12,13]. Following ischemic stroke, this maladaptive inflammatory response contributes directly to secondary neuronal injury, white matter damage, blood–brain barrier (BBB) disruption, and impaired synaptic remodeling, resulting in worse functional recovery in aged animals compared with younger counterparts [14,15,16].

Cellular senescence further amplifies cerebrovascular dysfunction in aging and represents a convergent pathological mechanism linking inflammation, vascular instability, and impaired repair. Senescent endothelial cells, pericytes, astrocytes, and microglia accumulate progressively within the aging brain and cerebrovasculature and adopt a senescence-associated secretory phenotype (SASP) characterized by sustained release of pro-inflammatory cytokines, chemokines, proteases, and growth factors. Original experimental studies have shown that senescent endothelial cells exhibit impaired nitric oxide signaling and increased permeability, directly contributing to BBB breakdown and microvascular dysfunction [17,18]. In parallel, accumulation of senescent glial cells has been shown to exacerbate neuroinflammation, impair synaptic homeostasis, and limit post-injury plasticity, thereby reducing responsiveness to endogenous repair mechanisms [19,20].

Mitochondrial dysfunction and metabolic decline represent additional hallmarks of aging-related cerebrovascular disease and critically influence tissue vulnerability and recovery capacity. Aging brain cells display reduced mitochondrial biogenesis, impaired oxidative phosphorylation, altered NAD^+^ metabolism, and defective mitochondrial quality control, leading to diminished energetic reserve and increased susceptibility to ischemic stress. Original studies have demonstrated that mitochondrial dysfunction in endothelial and neural cells promotes oxidative stress, inflammasome activation, and BBB disruption following ischemic injury [21,22,23]. Moreover, age-related deficits in mitochondrial function delay metabolic recovery after stroke and exacerbate neuronal loss and white matter injury [24,25].

**Figure 1:**
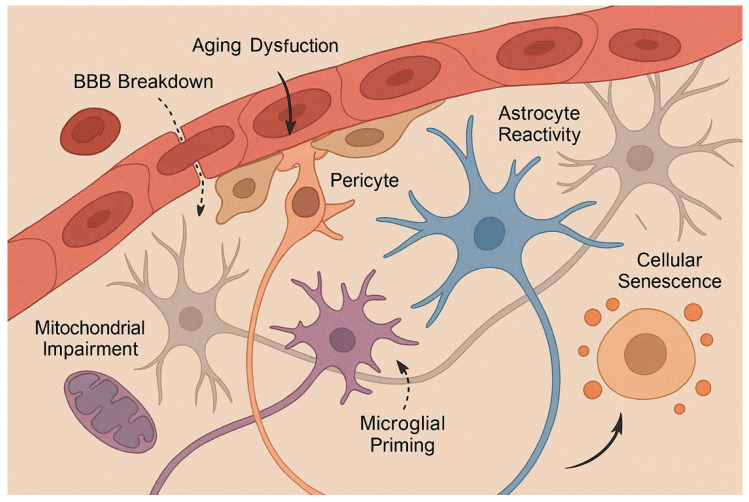
Conceptual schematic illustrating the major molecular and cellular mechanisms underlying aging-related cerebrovascular dysfunction. Aging promotes endothelial dysfunction, blood–brain barrier breakdown, pericyte loss, astrocyte reactivity, microglial priming, cellular senescence, and mitochondrial impairment within the neurovascular unit. These interconnected processes synergistically exacerbate neuroinflammation, impair cerebral perfusion and metabolic coupling, and increase vulnerability to ischemic injury while limiting regenerative capacity.

Importantly, aging-related cerebrovascular disease does not arise from isolated pathological processes but rather from the convergence of neuroinflammation, cellular senescence, metabolic insufficiency, BBB dysfunction, and progressive disintegration of the neurovascular unit. Experimental evidence indicates that these mechanisms interact bidirectionally, forming self-reinforcing pathological loops that accelerate vascular and neural injury in the aged brain [26,27,28,29]. This interconnected pathophysiological landscape explains why single-target interventions often show limited efficacy in elderly populations and provides a mechanistic foundation for the multimodal therapeutic strategies discussed in subsequent sections of this review (Figure 1).

## 4. Emerging Anti-Inflammatory and Immunomodulatory Strategies

Chronic neuroinflammation is a defining feature of aging-related cerebrovascular diseases and a major determinant of poor neurological outcomes in elderly patients. Unlike the transient inflammatory response observed in younger individuals, aging promotes a persistent, dysregulated inflammatory milieu characterized by sustained activation of innate immune pathways, impaired resolution mechanisms, and exaggerated responses to ischemic injury [11,30,31,32]. This maladaptive inflammatory state contributes to secondary neuronal damage, blood–brain barrier [BBB] breakdown, white matter degeneration, and impaired synaptic plasticity, making inflammation an attractive therapeutic target in the aging brain [33,34].

Aging-associated inflammation is driven in part by systemic inflammaging, a condition marked by elevated circulating levels of pro-inflammatory cytokines, chemokines, and damage-associated molecular patterns [DAMPs]. These signals prime cerebrovascular endothelial cells and resident immune cells, lowering the threshold for inflammatory activation following vascular injury [4,35,36]. In parallel, immunosenescence alters adaptive immune responses, leading to impaired immune regulation and prolonged innate immune activation, further amplifying neurovascular damage after stroke. Microglia play a central role in mediating age-related neuroinflammation and represent key regulators of cerebrovascular vulnerability in the aging brain [37,38].

With advancing age, microglia undergo phenotypic and functional remodeling, transitioning toward a so-called “primed” state characterized by heightened sensitivity to inflammatory stimuli, altered metabolic profiles, and exaggerated production of pro-inflammatory mediators, including tumor necrosis factor-α, interleukin-1β, interleukin-6, nitric oxide, and reactive oxygen species [39,40]. This primed phenotype is associated with transcriptional reprogramming toward inflammatory and complement-related pathways, reduced phagocytic efficiency, and impaired resolution of inflammatory responses [41].

In the context of cerebrovascular injury, aged microglia respond to ischemia with exaggerated and prolonged activation compared with their younger counterparts. Experimental studies have demonstrated that this age-dependent microglial hyper-responsiveness contributes to excessive synaptic pruning through complement-mediated mechanisms, increased neuronal apoptosis, and aggravated white matter injury, thereby impairing functional recovery after stroke [10,41]. In addition, primed microglia in the aged brain exhibit enhanced cross-talk with infiltrating peripheral immune cells, further amplifying neurovascular inflammation and blood–brain barrier disruption [11].

These findings underscore that maladaptive microglial activation is not merely a consequence but a driver of aging-related cerebrovascular pathology [10,11]. Accordingly, therapeutic strategies have shifted toward modulating microglial activation states rather than broadly suppressing inflammation, which may compromise essential repair processes. Among emerging targets, the NLRP3 inflammasome has gained particular attention as a central mediator linking aging, metabolic stress, and neuroinflammation [21,22,42,43,44,45,46,47,48,49,50,51].

Aging promotes NLRP3 priming through mitochondrial dysfunction, increased mitochondrial reactive oxygen species, impaired mitophagy, and accumulation of damage-associated molecular patterns, creating a permissive environment for exaggerated inflammasome activation following ischemic injury [21,45,48].

In experimental models of ischemic stroke, pharmacological inhibition or genetic suppression of NLRP3 signaling has been shown to attenuate microglial activation, reduce infarct volume, preserve blood–brain barrier integrity, and improve neurological outcomes, with particularly pronounced benefits observed in aged animals [12,52]. These findings support inflammasome inhibition as a mechanistically targeted strategy capable of dampening maladaptive microglial responses while preserving essential immune functions. Together, modulation of microglial priming and selective targeting of inflammasome signaling pathways represent promising avenues for mitigating aging-associated cerebrovascular inflammation and improving recovery in elderly patients.

**Figure 2:**
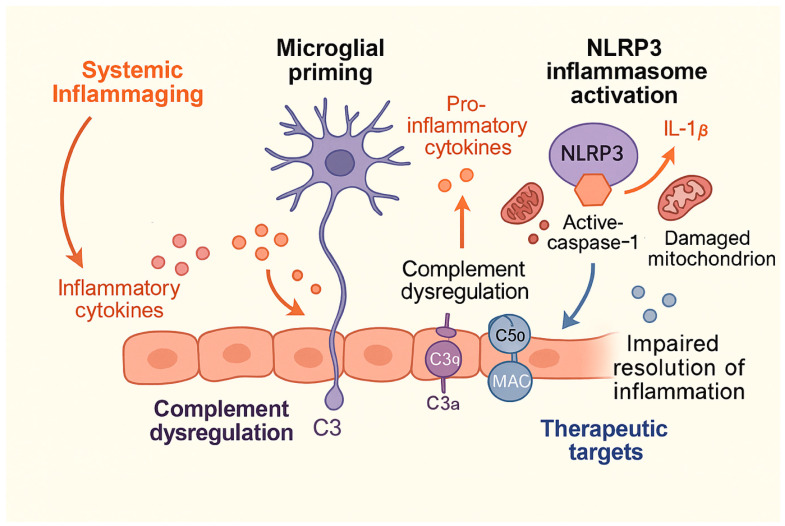
Immunomodulatory mechanisms and therapeutic targets in aging-related cerebrovascular inflammation. Aging is associated with systemic inflammaging, characterized by elevated circulating inflammatory mediators that prime cerebrovascular endothelial cells and resident immune cells. In the aged brain, microglia adopt a primed phenotype and respond to vascular injury with exaggerated production of pro-inflammatory cytokines. Mitochondrial dysfunction and accumulation of damage-associated molecular patterns promote activation of the NLRP3 inflammasome, leading to caspase-1 activation and increased interleukin-1β release. In parallel, age-related dysregulation of the complement cascade results in excessive generation of complement components and membrane attack complex formation, contributing to blood–brain barrier disruption and secondary tissue injury. Collectively, these processes impair resolution of inflammation and exacerbate neurovascular damage. Emerging immunomodulatory therapies aim to attenuate maladaptive inflammation by targeting microglial priming, inflammasome activation, complement signaling, and inflammatory cytokine pathways while preserving repair-associated immune functions.

Complement system activation represents another key inflammatory pathway implicated in age-related vascular brain injury. While complement signaling is essential for immune surveillance and synaptic remodeling, aging disrupts its regulation, leading to excessive complement activation and synapse loss [53,54,55]. In experimental stroke models, complement inhibition attenuates neuroinflammation and reduces secondary brain injury, suggesting potential therapeutic value, particularly in the aged brain where complement activity is often upregulated [22]. Beyond innate immune pathways, immunomodulatory strategies targeting cytokine signaling have also demonstrated promise. Interventions aimed at blocking interleukin-1 signaling or enhancing anti-inflammatory cytokines such as interleukin-10 have been shown to improve outcomes in experimental models of cerebrovascular injury, although age-specific efficacy and safety remain under investigation [56,57]. Importantly, complete suppression of inflammation may be detrimental, as controlled inflammatory responses are necessary for debris clearance and tissue repair. Thus, therapeutic approaches that promote resolution of inflammation rather than indiscriminate inhibition are increasingly favored (Figure 2).

Recent advances have highlighted the immunomodulatory potential of both cell-based and cell-free therapeutic strategies as promising approaches for mitigating aging-related cerebrovascular inflammation. Among these, mesenchymal stromal cells [MSCs] have emerged as potent modulators of innate and adaptive immune responses, exerting their therapeutic effects predominantly through paracrine mechanisms rather than direct cell replacement. MSCs secrete a diverse array of bioactive factors, including cytokines, chemokines, growth factors, and extracellular vesicles [EVs], that collectively suppress pro-inflammatory signaling, promote immune resolution, and enhance tissue repair [58,59].

In the context of neuroinflammation, MSCs have been shown to modulate microglial activation states, shifting microglia from a pro-inflammatory phenotype toward anti-inflammatory and reparative profiles. This polarization is associated with reduced production of tumor necrosis factor-α, interleukin-1β, and inducible nitric oxide synthase, alongside increased expression of anti-inflammatory mediators such as interleukin-10 and transforming growth factor-β [60,61]. Importantly, these immunomodulatory effects translate into preservation of blood–brain barrier integrity, attenuation of white matter injury, and improved functional recovery in experimental models of ischemic stroke.

Increasing evidence suggests that MSC-derived extracellular vesicles recapitulate many of the beneficial immunomodulatory properties of their parent cells. These EVs carry regulatory microRNAs, long non-coding RNAs, proteins, and lipids that directly influence gene expression and signaling pathways in recipient immune and neurovascular cells. In aged experimental models, MSC-derived EVs have been shown to suppress microglial pro-inflammatory signaling, reduce inflammasome activation, and enhance endogenous repair mechanisms, while avoiding the safety concerns associated with cell transplantation [62,63,64].

These immunoregulatory effects appear particularly relevant in aging contexts, where endogenous mechanisms responsible for resolution of inflammation are compromised by immunosenescence, chronic low-grade inflammation, and impaired intercellular communication. By restoring immune balance and promoting reparative signaling within the neurovascular unit, MSC-based and EV-based therapies offer a biologically rational strategy to counteract maladaptive inflammation and enhance recovery in aging-related cerebrovascular disease [65,66].

In summary, emerging anti-inflammatory and immunomodulatory strategies reflect a shift from broad immunosuppression toward precise targeting of age-altered immune pathways. By addressing microglial priming, inflammasome activation, complement dysregulation, and impaired inflammatory resolution, these approaches hold significant promise for improving outcomes in aging-related cerebrovascular diseases.

## 5. Senescence-Targeted Therapies in Aging-Related Cerebrovascular Disease

Cellular senescence has emerged as a central driver of aging-related cerebrovascular dysfunction and represents a promising therapeutic target for restoring vascular and neurovascular health in elderly individuals. Senescent cells accumulate progressively with age across multiple components of the cerebrovascular system, including endothelial cells, pericytes, astrocytes, microglia, and vascular smooth muscle cells, reflecting cumulative cellular stress, DNA damage, mitochondrial dysfunction, and impaired proteostasis [1,11]. Although senescence initially serves as a tumor-suppressive mechanism, its chronic persistence in aging tissues exerts deleterious effects on vascular and neural homeostasis.

Senescent cells undergo stable cell cycle arrest mediated by activation of the cyclin-dependent kinase inhibitor p53–p21CIP1 and p16INK4a–Rb pathways while remaining metabolically active and highly secretory. A defining feature of senescence is the acquisition of a senescence-associated secretory phenotype [SASP], characterized by sustained release of pro-inflammatory cytokines [e.g., interleukin-6, interleukin-1β], chemokines, growth factors, reactive oxygen species, and matrix-degrading enzymes such as matrix metalloproteinases [67,68]. In the cerebrovascular context, this maladaptive secretory profile promotes chronic low-grade inflammation, disrupts extracellular matrix integrity, and amplifies inflammatory signaling within the neurovascular unit.

Accumulating evidence indicates that senescent endothelial cells exhibit impaired nitric oxide bioavailability, increased oxidative stress, and reduced angiogenic capacity, directly contributing to vascular stiffness, microvascular rarefaction, and blood–brain barrier [BBB] breakdown [69,70]. Senescence of pericytes further destabilizes capillary networks and weakens BBB integrity, facilitating leakage of plasma-derived factors and infiltration of peripheral immune cells into the brain parenchyma. In parallel, senescent astrocytes and microglia amplify neuroinflammation through SASP-mediated signaling, impair synaptic homeostasis, and limit endogenous repair processes following ischemic or hypoperfusion-related injury [71,72,73].

This maladaptive secretory profile contributes to chronic inflammation, extracellular matrix remodeling, blood–brain barrier [BBB] breakdown, and impaired tissue repair [11]. Within the cerebral vasculature, senescent endothelial cells exhibit reduced nitric oxide production, impaired angiogenic signaling, increased oxidative stress, and altered mechanotransduction. These changes compromise cerebral blood flow regulation and promote microvascular rarefaction, thereby increasing susceptibility to ischemic injury [69]. Senescent pericytes further destabilize capillary networks and weaken BBB integrity, facilitating leakage of plasma proteins and infiltration of peripheral immune cells, processes strongly linked to cognitive decline and poor stroke outcomes in aging populations.

In the brain parenchyma, senescent astrocytes and microglia act as potent amplifiers of neuroinflammation through sustained senescence-associated secretory phenotype [SASP] signaling. Senescent microglia exhibit impaired phagocytic capacity, reduced clearance of cellular debris and myelin fragments, and exaggerated inflammatory responses to ischemic or metabolic stress. These cells show increased production of pro-inflammatory cytokines, reactive oxygen species, and complement factors, contributing to persistent synaptic pruning and neuronal injury [74,75]. In parallel, senescent astrocytes display altered metabolic coupling with neurons, reduced lactate shuttling, impaired antioxidant support, and increased release of inflammatory mediators and matrix-modifying enzymes, further destabilizing the neurovascular microenvironment [76].

This senescence-driven inflammatory amplification disrupts synaptic homeostasis, exacerbates white matter injury, and limits post-stroke plasticity and repair, particularly in the aged brain where compensatory mechanisms are diminished [71,77]. Importantly, the accumulation of senescent glial cells establishes a self-perpetuating cycle of inflammation, oxidative stress, and impaired repair that extends well beyond the acute phase of cerebrovascular injury.

These insights have catalyzed the development of senescence-targeted therapeutic strategies, broadly classified into senolytics and senomorphics. Senolytics are agents designed to selectively eliminate senescent cells by exploiting their dependence on anti-apoptotic and pro-survival pathways, including BCL-2/BCL-xL family signaling, PI3K/AKT pathways, and p53-related mechanisms. Pharmacological senolytics such as dasatinib and quercetin, navitoclax, and fisetin have demonstrated efficacy in reducing senescent cell burden, attenuating systemic and tissue-specific inflammation, and improving vascular function in aged experimental models [78,79,80]. In cerebrovascular contexts, senolytic treatment has been shown to improve endothelial function, enhance blood–brain barrier integrity, and reduce neuroinflammatory signaling, although direct evidence from aged stroke-specific models remains relatively limited and warrants further investigation [81]. In contrast, senomorphics aim to suppress the deleterious effects of the SASP without inducing senescent cell death. By modulating key inflammatory and metabolic pathways, including NF-κB, mTOR, and JAK/STAT signaling, senomorphics attenuate chronic inflammation, reduce extracellular matrix degradation, and limit secondary tissue damage while preserving potentially beneficial aspects of senescence, such as tumor suppression and wound containment [82]. Among these agents, rapamycin and related mTOR inhibitors have shown particular promise in improving vascular function, reducing neuroinflammation, and extending health span in aging models, including those relevant to cerebrovascular pathology [83,84]. These agents may be especially relevant for elderly patients, in whom widespread elimination of senescent cells could pose safety concerns related to impaired tissue repair or immune function.

Despite their therapeutic promise, senescence-targeted interventions face several translational challenges in aging-related cerebrovascular disease. Senescent cells are heterogeneous, context-dependent, and dynamically regulated, raising concerns that indiscriminate targeting may disrupt tissue homeostasis or impair wound healing. Furthermore, optimal timing, dosing, and combination strategies—particularly in relation to acute versus chronic phases of cerebrovascular injury—remain poorly defined. Nonetheless, accumulating evidence supports cellular senescence as a modifiable contributor to cerebrovascular aging, positioning both senolytics and senomorphics as promising components of future multimodal, precision-based therapeutic strategies.

## 6. Stem Cell-Based and Regenerative Approaches in Aging-Related Cerebrovascular Disease

Regenerative failure is a defining feature of cerebrovascular disease in the elderly, reflecting the cumulative impact of aging on vascular plasticity, neural repair, and immune resolution. Aging profoundly alters the brain’s intrinsic capacity to recover from ischemic or hypoperfusion-related injury by impairing endogenous neurogenesis, angiogenesis, synaptic remodeling, and remyelination, while simultaneously promoting chronic inflammation and senescence within the neurovascular niche [65,85]. In addition, age-related alterations in extracellular matrix composition, growth factor responsiveness, and metabolic support further limit reparative processes and contribute to incomplete functional recovery after cerebrovascular events. Within this context, stem cell-based therapies have attracted considerable attention as potential strategies to restore neurovascular integrity and enhance recovery in aging-related cerebrovascular disorders. Rather than replacing lost neurons or vascular cells directly, these approaches aim to modulate the hostile aging microenvironment by promoting angiogenesis, stabilizing blood–brain barrier function, suppressing maladaptive inflammation, and stimulating endogenous repair mechanisms [86,87].

Among the various cell types investigated, mesenchymal stromal cells [MSCs] have emerged as the most extensively studied candidates in experimental models of ischemic stroke, cerebral small vessel disease, and vascular cognitive impairment. MSCs derived from bone marrow, adipose tissue, or umbilical cord exhibit robust immunomodulatory, angiogenic, and trophic properties and display low immunogenicity, making them attractive for translational applications [59,88]. Importantly, accumulating evidence indicates that the therapeutic efficacy of MSCs relies predominantly on paracrine signaling rather than long-term engraftment or direct differentiation into neural or vascular cell types.

Through secretion of cytokines, growth factors, chemokines, and extracellular vesicles, MSCs modulate microglial and macrophage activation, suppress pro-inflammatory cytokine production, enhance angiogenesis, and promote synaptic and white matter remodeling. These paracrine effects are particularly relevant in aging contexts, where endogenous repair pathways are blunted and immune resolution is compromised [58,89]. MSCs secrete a broad repertoire of growth factors, cytokines, and extracellular vesicles that modulate inflammation, enhance endothelial survival, and promote neurovascular remodeling [58]. In aged experimental models of ischemic stroke, MSC administration has been shown to reduce infarct volume, preserve blood–brain barrier integrity, and improve functional recovery, although the magnitude of benefit is often attenuated compared with younger counterparts. This reduced efficacy reflects the hostile aging microenvironment, characterized by chronic inflammation, oxidative stress, and impaired responsiveness to growth factor signaling [65]. Nevertheless, MSC-mediated immunomodulation—particularly suppression of pro-inflammatory microglial activation and promotion of anti-inflammatory phenotypes—remains a key mechanism of benefit in the aged brain.

Neural stem and progenitor cells [NSPCs] represent another promising regenerative strategy for aging-related cerebrovascular disease, given their intrinsic capacity to differentiate into neurons, astrocytes, and oligodendrocytes and to integrate into existing neural circuits. In the adult brain, NSPCs residing in neurogenic niches such as the subventricular zone and hippocampal dentate gyrus contribute to limited neuronal replacement and circuit remodeling. However, aging profoundly impairs NSPC biology, leading to reduced stem cell numbers, diminished proliferative capacity, mitochondrial dysfunction, and epigenetic alterations that skew differentiation toward gliogenesis at the expense of neurogenesis [90,91]. These age-related changes constrain endogenous repair and contribute to cognitive decline and impaired recovery after cerebrovascular injury.

Exogenous transplantation of NSPCs has been shown to enhance neuroplasticity, promote white matter repair, and improve cognitive and functional outcomes in experimental models of ischemic stroke and vascular injury. These benefits are mediated through a combination of limited cell replacement and, more prominently, paracrine mechanisms that support angiogenesis, modulate inflammation, and stimulate endogenous progenitor populations [86,92]. Nevertheless, age-related reductions in graft survival, migration, and synaptic integration pose significant challenges, particularly in aged hosts with hostile inflammatory and metabolic microenvironments [65].

Despite encouraging preclinical data, several barriers hinder the clinical translation of stem cell-based therapies in aging populations. Aging alters stem cell intrinsic properties, including proliferative capacity, mitochondrial function, and genomic stability, while the aged neurovascular niche exhibits reduced responsiveness to trophic and regenerative cues due to chronic inflammation, senescence, and extracellular matrix remodeling [70,85]. These limitations have driven the exploration of combinatorial strategies aimed at modifying both the therapeutic cells and the host environment.

**Figure 3:**
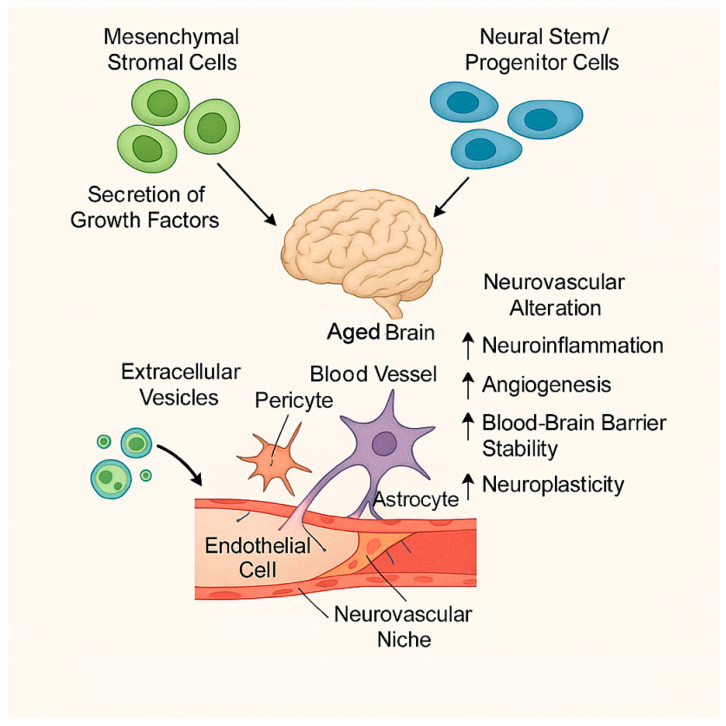
Stem cell-based and regenerative strategies in aging-related cerebrovascular disease. Schematic representation of regenerative approaches targeting neurovascular dysfunction in the aging brain. Mesenchymal stromal cells and neural stem/progenitor cells exert therapeutic effects predominantly through paracrine mechanisms, including the secretion of growth factors and extracellular vesicles. These signals act on multiple components of the neurovascular niche to modulate neuroinflammation, promote angiogenesis, stabilize blood–brain barrier integrity, and enhance neuroplasticity. In aging-related cerebrovascular disease, alterations in the neurovascular microenvironment limit endogenous repair capacity, and stem cell-based interventions aim to restore vascular and neural resilience through coordinated modulation of inflammatory and regenerative pathways.

Emerging approaches include preconditioning of stem cells to enhance stress resistance and paracrine potency, genetic modification to augment trophic or anti-inflammatory signaling, and combination therapies integrating stem cells with anti-inflammatory, metabolic, or senescence-targeted interventions [59,87]. Collectively, stem cell-based and regenerative strategies represent a promising yet complex avenue for treating aging-related cerebrovascular diseases. Continued refinement of delivery methods, optimization for aged microenvironments, and rigorous evaluation in age-appropriate experimental and clinical models will be essential for successful translation (Figure 3).

## 7. Extracellular Vesicles and RNA-Based Therapies in Aging-Related Cerebrovascular Disease

Intercellular communication within the neurovascular unit is profoundly altered by aging, contributing to chronic low-grade inflammation, blood–brain barrier [BBB] dysfunction, impaired neurovascular coupling, and reduced reparative capacity following cerebrovascular injury. Aging disrupts coordinated signaling among the NVU cells leading to maladaptive inflammatory amplification and compromised vascular integrity [2,3]. These alterations create a permissive environment for sustained injury propagation and limit endogenous repair in elderly individuals.

Extracellular vesicles [EVs], including exosomes and microvesicles, have emerged as central mediators of intercellular communication within the neurovascular unit. EVs transport a diverse array of bioactive cargo—such as microRNAs [miRNAs], long non-coding RNAs [lncRNAs], messenger RNAs, proteins, lipids, and metabolites—that regulate gene expression, signal transduction, and cellular phenotype in recipient cells [93,94]. In the brain, EV-mediated signaling plays critical roles in maintaining vascular homeostasis, modulating immune responses, and coordinating metabolic and synaptic function.

Accumulating evidence indicates that aging profoundly alters EV biogenesis, release, and molecular composition. Senescent endothelial cells, astrocytes, and microglia secrete EVs enriched in pro-inflammatory cytokines, adhesion molecules, and regulatory RNAs that promote endothelial activation, microglial priming, and BBB disruption [95,96]. These age-associated EV signatures propagate inflammatory signaling across the neurovascular unit and contribute to increased cerebrovascular vulnerability and impaired recovery after ischemic injury. Conversely, therapeutic manipulation of EVs and their RNA cargo has emerged as a promising, cell-free regenerative strategy. EVs derived from stem and progenitor cells carry regulatory miRNAs and lncRNAs that modulate angiogenesis, immune resolution, mitochondrial function, and synaptic plasticity. In experimental models of cerebrovascular disease, administration of engineered or stem cell-derived EVs has been shown to enhance vascular remodeling, stabilize BBB integrity, suppress maladaptive inflammation, and improve functional outcomes [58,62]. Importantly, these approaches bypass limitations associated with cell transplantation, including poor engraftment, immune rejection, and tumorigenic risk, making them particularly attractive for elderly patients.

In the aging brain and vasculature, EV profiles are markedly altered. Senescent endothelial cells, astrocytes, and microglia release EVs enriched in pro-inflammatory cytokines, adhesion molecules, and regulatory RNAs that amplify inflammaging and endothelial dysfunction. These EVs promote microglial priming, impair tight junction integrity, and disrupt neurovascular coupling, thereby exacerbating susceptibility to ischemic injury [95,96]. Conversely, EVs derived from young or healthy cells convey protective signals that support angiogenesis, mitochondrial function, and synaptic maintenance, underscoring the functional importance of EV cargo composition. Among EV cargos, miRNAs have received particular attention as regulators of cerebrovascular homeostasis. Specific miRNAs, such as miR-126, miR-132, and miR-210, play critical roles in endothelial survival, angiogenesis, and BBB stability [97,98,99,100,101]. Aging is associated with dysregulation of these miRNAs, leading to impaired vascular repair and increased inflammation. Experimental delivery of EVs enriched in pro-angiogenic or anti-inflammatory miRNAs has been shown to enhance vascular remodeling, reduce infarct size, and improve functional recovery in models of ischemic stroke, including aged animals [102,103,104]. Long non-coding RNAs and circular RNAs carried by EVs further modulate gene expression networks involved in inflammation, oxidative stress, and cell survival. Although their roles in aging-related cerebrovascular disease are only beginning to be elucidated, emerging data suggest that EV-associated lncRNAs can influence endothelial senescence, microglial activation states, and astrocyte reactivity, highlighting their potential as therapeutic targets or biomarkers [105].

Stem cell-derived EVs represent a particularly attractive therapeutic platform. EVs isolated from mesenchymal stromal cells or neural stem/progenitor cells recapitulate many of the beneficial effects of their parent cells, including immunomodulation, promotion of angiogenesis, and enhancement of neuroplasticity, while avoiding risks associated with cell transplantation. In aging contexts, these EVs have been shown to suppress microglial pro-inflammatory signaling, preserve BBB integrity, and improve neurological outcomes after stroke [81]. Importantly, EVs can cross the BBB, enabling systemic delivery and broad distribution within the brain.

Beyond extracellular vesicle-based approaches, direct RNA-based therapeutic strategies are gaining substantial momentum as precision tools for modulating pathogenic gene expression in aging-related cerebrovascular disease. These approaches include synthetic microRNA [miRNA] mimics or inhibitors [antagomiRs], antisense oligonucleotides [ASOs], and RNA interference [RNAi] technologies, all of which enable sequence-specific regulation of target transcripts with high molecular precision [106,107]. Advances in RNA chemistry and delivery systems have substantially improved the stability, specificity, and safety profiles of these therapeutics, accelerating their translational potential.

In the context of cerebrovascular aging, RNA-based tools offer the ability to selectively target age-associated inflammatory pathways, senescence regulators, and metabolic genes that drive neurovascular dysfunction. For example, modulation of miRNAs involved in endothelial inflammation, microglial activation, and mitochondrial homeostasis—such as miR-155, miR-21, and miR-126—has been shown to influence blood–brain barrier integrity, inflammatory signaling, and angiogenic capacity in experimental models [108,109]. Similarly, RNA-based suppression of senescence-associated regulators and inflammatory transcriptional programs offers a strategy to attenuate chronic SASP signaling without eliminating senescent cells.

**Figure 4:**
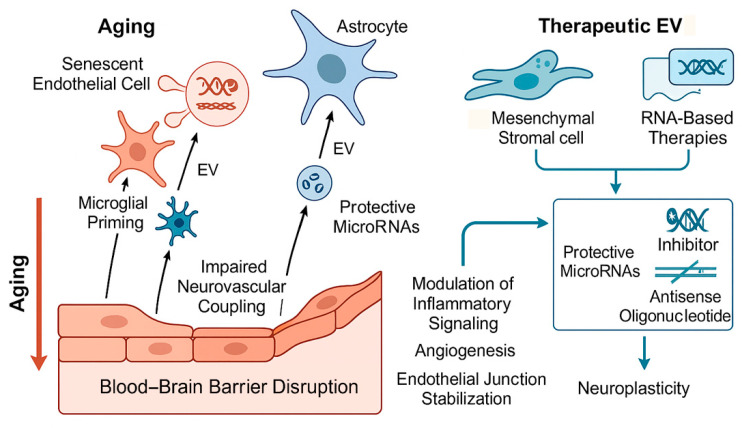
Extracellular vesicle- and RNA-based therapeutic mechanisms in aging-related cerebrovascular disease. Single-panel schematic illustrating how aging alters extracellular vesicle (EV)-mediated communication within the neurovascular unit and how EV- and RNA-based interventions may restore neurovascular function. In the aging brain, senescent endothelial cells and primed microglia release EVs carrying pro-inflammatory cargo that amplifies neuroinflammation, disrupts neurovascular coupling, and compromises blood–brain barrier integrity. Astrocyte-mediated signaling is similarly altered, contributing to impaired vascular–neuronal communication. Therapeutic EVs derived from mesenchymal stromal cells deliver protective microRNAs and other regulatory molecules that modulate inflammatory signaling, promote angiogenesis, stabilize endothelial junctions, and support neuroplasticity. Complementary RNA-based therapies, including microRNA mimics, inhibitors, and antisense oligonucleotides, aim to selectively reprogram maladaptive gene expression networks associated with cerebrovascular aging, offering a cell-free precision approach to neurovascular repair.

RNA interference and antisense approaches also enable targeting of metabolic and mitochondrial pathways that become dysregulated with aging, including genes involved in oxidative stress responses, NAD^+^ metabolism, and inflammasome activation. By reprogramming maladaptive gene expression profiles, RNA-based therapeutics can theoretically restore neurovascular homeostasis while minimizing off-target systemic effects [110] (Figure 4). Importantly, the modular nature of RNA therapeutics allows rapid adaptation to emerging molecular targets identified through omics and biomarker studies, aligning well with precision medicine frameworks.

Despite their promise, several challenges remain for the application of RNA-based therapies in aging-related cerebrovascular disease, including efficient delivery across the blood–brain barrier, cell-type specificity, and long-term safety in elderly populations. Nonetheless, continued advances in delivery vectors, chemical stabilization, and targeting strategies position RNA-based therapeutics as a highly flexible and mechanistically grounded approach for addressing the molecular complexity of cerebrovascular aging. However, efficient delivery to the aging brain, avoidance of off-target effects, and long-term safety remain key challenges [111]. Collectively, EV- and RNA-based therapies represent a paradigm shift toward precision, cell-free interventions that directly target the molecular drivers of cerebrovascular aging. By restoring adaptive intercellular communication and reprogramming maladaptive inflammatory and senescent pathways, these strategies hold substantial promise for improving outcomes in aging-related cerebrovascular diseases.

## 8. Targeting the Neurovascular Unit and Blood–Brain Barrier Integrity

The NVU is a highly integrated functional ensemble that regulates cerebral blood flow, metabolic coupling, synaptic activity, and blood–brain barrier [BBB] integrity. Tight coordination among these cellular elements ensures precise matching of neuronal energy demands with vascular supply and maintains the selective permeability of the cerebral microvasculature [3,112]. Disruption of NVU signaling therefore has profound consequences for brain homeostasis and function.

Aging progressively disrupts the coordinated signaling within the NVU through endothelial dysfunction, pericyte loss, astrocyte reactivity, microglial priming, and remodeling of the extracellular matrix. These changes lead to increased BBB permeability, impaired neurovascular coupling, altered cerebral autoregulation, and reduced metabolic support to neurons, collectively increasing vulnerability to ischemic and hypoxic injury [2,113]. Importantly, age-related BBB breakdown has been shown to precede overt neurodegeneration and cognitive decline, highlighting its causal role in cerebrovascular and neurodegenerative pathology.

BBB dysfunction in aging is both a driver and a consequence of neuroinflammation and vascular pathology. Increased permeability facilitates the entry of plasma proteins, peripheral immune cells, and inflammatory mediators into the brain parenchyma, amplifying microglial activation and astrocytic reactivity. In turn, chronic neuroinflammation and oxidative stress further compromise endothelial tight junctions and pericyte–endothelial interactions, establishing a self-reinforcing cycle of NVU dysfunction [114,115].

Given the central role of NVU disintegration in aging-related cerebrovascular disease, therapeutic strategies aimed at restoring NVU integrity have emerged as a critical objective. Approaches that stabilize endothelial function, preserve pericyte coverage, modulate astrocytic and microglial responses, and reinforce extracellular matrix homeostasis offer the potential to simultaneously improve BBB integrity, reduce neuroinflammation, and enhance neurovascular resilience in the aging brain [112,116].

Endothelial dysfunction is a primary contributor to BBB breakdown in aging. Aged endothelial cells exhibit reduced expression of tight junction proteins, altered transporter activity, increased oxidative stress, and heightened inflammatory signaling. These changes promote paracellular leakage, facilitate immune cell infiltration, and impair cerebral autoregulation. Experimental studies have demonstrated that age-related BBB permeability precedes cognitive decline and exacerbates ischemic injury, underscoring the causal role of endothelial dysfunction in neurovascular aging [113].

Pericytes play a critical role in maintaining BBB integrity and capillary stability, yet they are particularly vulnerable to aging. Age-dependent pericyte loss and functional impairment lead to capillary rarefaction, reduced cerebral blood flow, and increased BBB permeability. Genetic or pharmacological preservation of pericyte function has been shown to attenuate vascular leakage and neurodegeneration in experimental models, highlighting pericytes as a promising therapeutic target [114]. Astrocytes constitute another essential component of the NVU by regulating endothelial tight junctions, ion homeostasis, and metabolic support to neurons. In aging, astrocytes increasingly adopt reactive phenotypes characterized by altered calcium signaling, reduced metabolic coupling, and increased secretion of inflammatory mediators. These changes compromise BBB stability and amplify neuroinflammatory cascades following cerebrovascular injury [117]. Therapeutic strategies aimed at restoring astrocytic homeostatic functions may therefore contribute to BBB repair and improved neurovascular resilience.

Microglial activation further exacerbates BBB dysfunction in aging by releasing cytokines, proteases, and reactive oxygen species that degrade tight junctions and extracellular matrix components. Aging-associated microglial priming leads to exaggerated inflammatory responses following ischemia or hypoperfusion, accelerating secondary vascular damage. Targeted modulation of microglial activation states has been shown to preserve BBB integrity and reduce neurovascular injury in preclinical models [10].

Several therapeutic approaches aimed at restoring NVU and BBB integrity are currently under investigation. These include pharmacological agents targeting endothelial nitric oxide signaling, angiopoietin–Tie2 pathways, and Wnt/β-catenin signaling, all of which play key roles in vascular stability and BBB maintenance. In addition, anti-inflammatory therapies, senescence-targeted interventions, and stem cell-derived extracellular vesicles have demonstrated capacity to stabilize BBB function indirectly by modulating the aging neurovascular microenvironment [3].

Importantly, effective restoration of blood–brain barrier [BBB] integrity in aging-related cerebrovascular disease is unlikely to be achieved through single-target interventions and will instead require combinatorial strategies that simultaneously address dysfunction across multiple components of the neurovascular unit. Aging-related BBB breakdown arises from the convergence of endothelial barrier failure, pericyte degeneration, astrocyte reactivity, microglial priming, and extracellular matrix remodeling, each of which contributes independently and synergistically to vascular instability and neuroinflammation [112,118].

Therapeutic approaches that stabilize endothelial tight junction signaling, preserve pericyte–endothelial interactions, normalize astrocytic metabolic and homeostatic functions, and modulate microglial activation states have the potential to interrupt self-reinforcing cycles of inflammation and barrier disruption. Preclinical studies indicate that targeting multiple NVU components concurrently results in greater preservation of BBB integrity, improved neurovascular coupling, and enhanced resistance to ischemic and inflammatory injury compared with single-pathway modulation [3,73].

**Figure 5:**
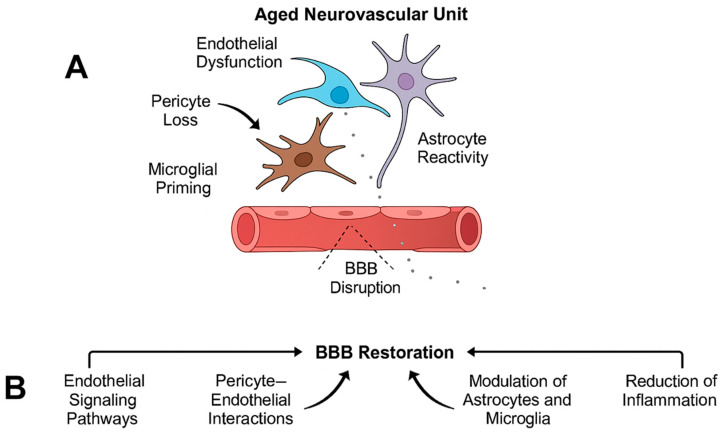
Neurovascular unit dysfunction and therapeutic restoration of blood–brain barrier integrity in aging-related cerebrovascular disease. (**A**) Single-panel schematic illustrating age-related alterations within the neurovascular unit that culminate in blood–brain barrier (BBB) disruption. Aging promotes endothelial dysfunction, pericyte loss, astrocyte reactivity, and microglial priming, leading to impaired tight junction integrity, increased vascular permeability, and leakage of blood-derived factors into the brain parenchyma. These changes amplify neuroinflammation and disrupt neurovascular coupling, thereby increasing susceptibility to cerebrovascular injury. (**B**) Therapeutic strategies aimed at restoring BBB integrity target endothelial signaling pathways, reinforce pericyte–endothelial interactions, modulate astrocytic and microglial activation states, and reduce chronic inflammation. Collectively, these interventions seek to re-establish neurovascular unit homeostasis and enhance cerebrovascular resilience in the aging brain.

By addressing endothelial dysfunction, pericyte loss, astrocyte reactivity, and microglial priming within an integrated therapeutic framework, NVU-centered strategies offer a mechanistically grounded approach to reducing cerebrovascular vulnerability and improving neurological outcomes in elderly patients. Such multidimensional interventions align closely with emerging precision medicine paradigms and may prove essential for translating experimental advances into effective, age-adapted therapies for cerebrovascular disease [115,119] (Figure 5).

## 9. Metabolic and Mitochondrial Interventions in Aging-Related Cerebrovascular Disease

Metabolic insufficiency and mitochondrial dysfunction are central hallmarks of cerebrovascular aging and critically influence susceptibility to ischemic injury, blood–brain barrier [BBB] integrity, and post-injury recovery. Aging is associated with a progressive decline in metabolic flexibility, characterized by reduced capacity to adapt to energetic stress, impaired mitochondrial biogenesis, and dysregulated substrate utilization across neurovascular unit cell types [117,120]. These changes are accompanied by increased mitochondrial reactive oxygen species production, defective mitophagy, and accumulation of damaged mitochondria, collectively compromising cellular homeostasis.

Within the aging brain, mitochondrial dysfunction affects the NVU-disrupting processes essential for cerebrovascular integrity. Impaired mitochondrial oxidative phosphorylation and reduced NAD^+^ availability weaken endothelial nitric oxide signaling, destabilize tight junction complexes, and promote BBB permeability [70,118]. In parallel, metabolic stress in astrocytes and microglia alters immunometabolic signaling, favoring pro-inflammatory activation states that further exacerbate vascular dysfunction and neuronal injury [119]. Given the brain’s exceptionally high and continuous energy demands, even modest age-related perturbations in mitochondrial function and metabolic regulation can have profound consequences for cerebrovascular health. Reduced energetic reserve limits the ability of the aging brain to withstand ischemic or hypoxic stress, delays recovery of ionic and metabolic gradients, and amplifies secondary injury cascades following stroke [120,121]. Taken together, these observations position metabolic and mitochondrial dysfunction as upstream, mechanistically actionable drivers of aging-related cerebrovascular vulnerability and poor neurological outcome.

Mitochondrial dysfunction in aging affects multiple cell types within the NVU. Aging mitochondria exhibit reduced oxidative phosphorylation efficiency, increased production of reactive oxygen species, impaired calcium handling, and altered dynamics of fission and fusion. These changes promote endothelial dysfunction, exacerbate neuroinflammation, and sensitize the brain to ischemic damage [120]. In cerebral endothelial cells, mitochondrial impairment disrupts nitric oxide signaling and tight junction maintenance, contributing directly to BBB breakdown.

Aging is also associated with a decline in nicotinamide adenine dinucleotide [NAD^+^] availability, a critical cofactor for mitochondrial respiration, DNA repair, and sirtuin-mediated metabolic regulation. Reduced NAD^+^ levels impair mitochondrial function and amplify inflammatory signaling, creating a feed-forward loop of metabolic decline and vascular dysfunction. Preclinical studies have demonstrated that NAD^+^ augmentation through precursors such as nicotinamide riboside or nicotinamide mononucleotide improves endothelial function, reduces oxidative stress, and enhances cerebrovascular resilience in aged models [122].

Mitochondrial-targeted antioxidants represent another promising therapeutic approach. Agents such as MitoQ and SS-31 selectively accumulate within mitochondria and neutralize reactive oxygen species at their source. In experimental models of aging and ischemic stroke, these compounds have been shown to preserve mitochondrial integrity, attenuate neuroinflammation, and improve neurological outcomes [123]. Importantly, mitochondrial antioxidants may offer advantages over conventional antioxidants, which have shown limited efficacy in clinical trials due to poor cellular targeting.

Metabolic reprogramming strategies that enhance cellular energy efficiency and stress resistance have also gained interest. Activation of AMP-activated protein kinase [AMPK] and sirtuin pathways promotes mitochondrial biogenesis, autophagy, and metabolic adaptation under energetic stress. Pharmacological agents such as metformin and caloric restriction mimetics have been shown to improve vascular function and reduce inflammation in aging models, although their specific effects on cerebrovascular outcomes require further investigation [124].

Beyond energy metabolism, mitochondrial dysfunction influences immune responses in aging-related cerebrovascular disease. Damaged mitochondria release mitochondrial DNA and other damage-associated molecular patterns that activate innate immune pathways, including the NLRP3 inflammasome. This immunometabolic coupling links mitochondrial failure directly to chronic neuroinflammation and BBB disruption [48].

Therapeutic strategies that restore mitochondrial quality control through enhanced mitophagy or reduction in mitochondrial stress may therefore exert dual metabolic and anti-inflammatory benefits in aging-related cerebrovascular disease. Impaired mitophagy in aging leads to the accumulation of dysfunctional mitochondria, increased mitochondrial reactive oxygen species production, and release of mitochondrial damage-associated molecular patterns that activate innate immune pathways, including the NLRP3 inflammasome [48,125]. Experimental enhancement of mitophagy or pharmacological reduction in mitochondrial stress has been shown to attenuate neuroinflammation, preserve endothelial function, and improve blood–brain barrier integrity in models of vascular and metabolic aging [126,127].

**Figure 6:**
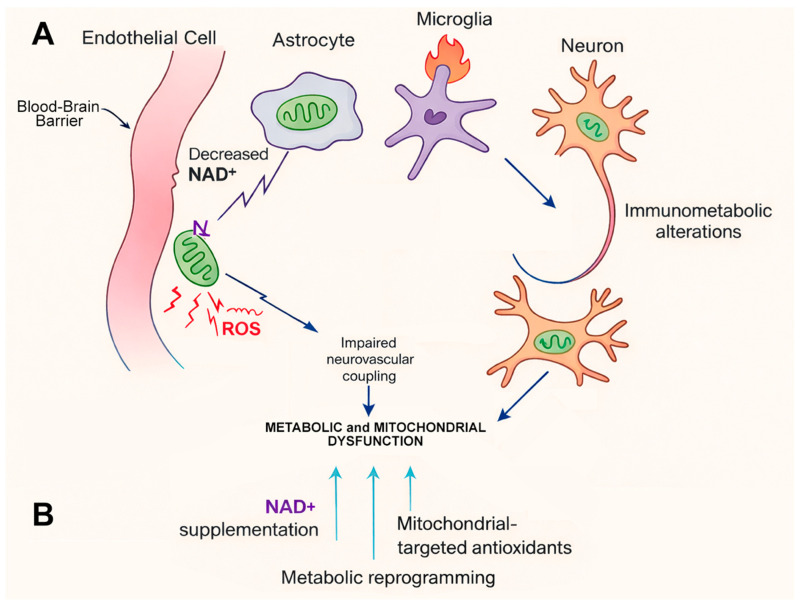
Metabolic and mitochondrial dysfunction as therapeutic targets in aging-related cerebrovascular disease. (**A**) Schematic illustration showing how aging-associated metabolic decline and mitochondrial dysfunction contribute to neurovascular injury. In cerebrovascular endothelial cells, reduced NAD^+^ availability and impaired oxidative phosphorylation lead to increased mitochondrial reactive oxygen species (ROS) production, disrupting endothelial signaling and compromising blood–brain barrier integrity. Astrocytes exhibit mitochondrial dysfunction and altered metabolic support, contributing to impaired neurovascular coupling. In parallel, metabolic stress in microglia drives immunometabolic reprogramming toward a pro-inflammatory phenotype, further amplifying chronic neuroinflammation and neuronal injury. Neurons experience reduced mitochondrial efficiency and energy failure, exacerbating synaptic dysfunction and vulnerability to ischemic stress. (**B**) Therapeutic strategies targeting mitochondrial health—including NAD^+^ supplementation, mitochondrial-targeted antioxidants, and metabolic reprogramming via energy-sensing pathways—aim to restore mitochondrial function, reduce oxidative stress, improve neurovascular coupling, and enhance cerebrovascular resilience in the aging brain.

Collectively, metabolic and mitochondrial interventions address fundamental upstream drivers of cerebrovascular aging rather than downstream consequences alone. By restoring cellular energy homeostasis, reducing oxidative and mitochondrial stress, and modulating immunometabolic signaling pathways, these strategies directly target the convergence point of vascular dysfunction, chronic inflammation, and impaired repair [117,118]. Importantly, metabolic reprogramming through NAD^+^ restoration, AMPK activation, and mitochondrial quality control enhancement has the potential to improve neurovascular resilience, expand therapeutic windows, and enhance recovery following cerebrovascular injury in the aging brain [70,120]. These findings suggest potential metabolic and mitochondrial therapies as broadly acting interventions capable of modifying disease trajectory and improving outcomes in aging-related cerebrovascular diseases (Figure 6).

## 10. Chronobiology- and Circadian-Based Therapeutic Approaches

Circadian rhythms are endogenous, approximately 24 h oscillations that regulate a wide range of physiological processes, including vascular tone, blood pressure, metabolism, immune function, and cellular repair. These rhythms are orchestrated by the central circadian pacemaker located in the suprachiasmatic nucleus [SCN] of the hypothalamus and by peripheral molecular clocks expressed in virtually all cell types, including the NVU [128,129]. Tight synchronization between central and peripheral clocks ensures temporal coordination of neurovascular, metabolic, and immune functions essential for cerebrovascular homeostasis.

Aging is associated with progressive circadian disruption, characterized by reduced rhythm amplitude, phase advances or delays, fragmented activity–rest cycles, and impaired entrainment to environmental cues. At the cellular level, aging leads to dampened oscillations of clock gene expression and impaired synchronization between central and peripheral clocks, resulting in temporal disorganization of physiological processes [130,131].

Increasing evidence indicates that this circadian dysregulation is a critical, yet underappreciated, contributor to aging-related cerebrovascular disease, influencing stroke risk, vascular inflammation, and recovery capacity. At the molecular level, circadian rhythms are generated by transcriptional–translational feedback loops involving core clock genes, including *BMAL1* and *CLOCK*, which drive the rhythmic expression of *PER* and *CRY* genes. The PER and CRY proteins subsequently inhibit their own transcription by suppressing BMAL1/CLOCK activity, generating self-sustained oscillations. Additional stabilizing loops involve nuclear receptors such as REV-ERBα/β and RORs, which fine-tune clock amplitude and phase [128,132]. Importantly, core clock components directly regulate the expression of genes involved in vascular homeostasis, oxidative stress responses, metabolism, and inflammation. In endothelial cells, circadian clock disruption impairs nitric oxide signaling, promotes oxidative stress, and alters vascular reactivity.

In immune and glial cells, circadian misalignment enhances pro-inflammatory gene expression and blunts resolution pathways, linking clock dysfunction to chronic neuroinflammation and blood–brain barrier instability [133,134]. These findings establish circadian regulation as a fundamental layer of control over neurovascular integrity and inflammatory balance, particularly relevant in the aging brain.

In aging, expression and oscillatory regulation of core clock genes are dampened, leading to loss of temporal organization of vascular and immune functions [130]. Disruption of *BMAL1* signaling, in particular, has been shown to accelerate vascular aging, promote endothelial dysfunction, and increase susceptibility to ischemic injury [133]. Circadian control of cerebrovascular physiology is especially relevant to stroke risk and outcome. Blood pressure, platelet aggregability, fibrinolytic activity, and cerebral blood flow all exhibit pronounced circadian variation, with a peak in ischemic stroke incidence occurring during the early morning hours. Aging exaggerates these vulnerabilities by impairing circadian regulation of vascular tone and coagulation pathways [135]. Moreover, experimental disruption of circadian rhythms worsens infarct size, blood–brain barrier breakdown, and neuroinflammation following ischemic stroke, highlighting a causal link between circadian integrity and cerebrovascular resilience.

Circadian regulation also intersects closely with neuroinflammation and immunometabolism. Microglial activation states and cytokine production exhibit circadian oscillations, which are blunted with aging. Loss of circadian control promotes sustained pro-inflammatory signaling and impairs resolution of inflammation after injury. Similarly, astrocytic metabolic support to neurons and endothelial cells is temporally regulated and becomes dysregulated in aged brains, contributing to impaired neurovascular coupling [136]. These findings position circadian disruption as a unifying mechanism linking aging, inflammation, metabolism, and vascular dysfunction.

Chronobiology-informed therapeutic strategies, collectively referred to as chronotherapy, aim to restore circadian alignment or exploit time-of-day-dependent variations in drug efficacy and toxicity. In the context of cerebrovascular disease, timing of antihypertensive, antiplatelet, and anticoagulant therapies has been shown to influence vascular outcomes, with evening or bedtime dosing improving blood pressure control and reducing cardiovascular risk in some elderly populations [137]. Although less studied in stroke, these principles are increasingly relevant for aging-related cerebrovascular prevention.

Beyond pharmacological timing, direct interventions targeting the circadian system itself are gaining increasing interest as strategies to restore temporal organization of neurovascular and immune functions in aging. These approaches include light-based therapies, melatonin supplementation, and behavioral interventions such as time-restricted feeding, structured sleep–wake schedules, and sleep optimization. Such interventions aim to reinforce central–peripheral clock synchronization and re-establish rhythmic regulation of metabolism, inflammation, and vascular tone, which are progressively disrupted with age [138,139].

Melatonin, a pineal-derived circadian hormone, is of particular interest in aging-related cerebrovascular disease due to its combined chronobiotic, antioxidant, and anti-inflammatory properties. Melatonin secretion declines markedly with advancing age, contributing to circadian fragmentation, sleep disturbances, and increased vulnerability to oxidative and inflammatory stress. In experimental models of ischemic stroke, melatonin administration has been shown to reduce infarct volume, preserve blood–brain barrier integrity, attenuate microglial activation, and improve neurological outcomes through mechanisms involving mitochondrial protection, free radical scavenging, and suppression of pro-inflammatory signaling pathways [140,141].

Behavioral circadian interventions, including time-restricted feeding and sleep optimization, also exert profound effects on metabolic and immune pathways relevant to cerebrovascular health. Restricting food intake to defined circadian windows improves metabolic efficiency, reduces systemic inflammation, and enhances mitochondrial function, even without caloric restriction. These effects may be particularly beneficial in elderly individuals with metabolic comorbidities that exacerbate cerebrovascular risk [142,143].

**Figure 7:**
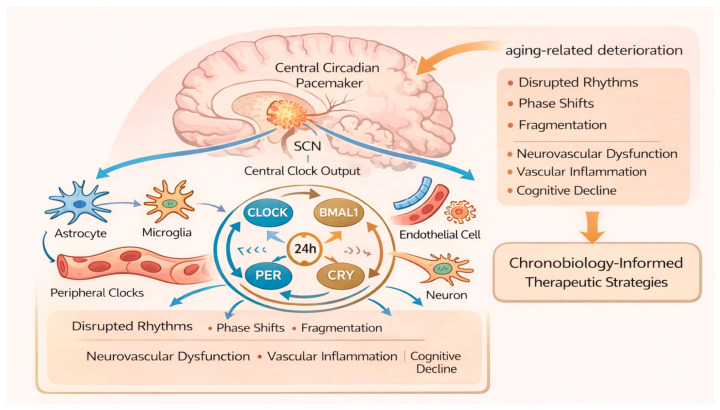
Circadian dysregulation as a contributor to aging-related cerebrovascular disease and a target for chronotherapy. Single-panel schematic illustrating the impact of age-associated circadian disruption on cerebrovascular function and neuroinflammation. Dysregulation of the molecular clock, exemplified by altered *BMAL1* signaling and impaired synchronization between central and peripheral clocks, disrupts temporal control of vascular tone, endothelial function, and neurovascular coupling. Circadian misalignment promotes astrocyte and microglial activation, exacerbates chronic neuroinflammation, and increases neuronal vulnerability to metabolic and ischemic stress, collectively contributing to cerebrovascular disease progression. Chronobiology-based therapeutic strategies—including light therapy, melatonin supplementation, sleep optimization, and time-dependent pharmacological interventions—aim to restore circadian alignment, dampen inflammation, and improve neurovascular resilience in the aging brain.

Taken together, circadian dysregulation represents a modifiable and mechanistically relevant contributor to aging-related cerebrovascular disease. Integrating chronobiological principles into therapeutic strategies offers a novel dimension of precision medicine, enabling optimization of treatment timing, reinforcement of endogenous repair mechanisms, and reduction in vascular and inflammatory risk in elderly individuals. As circadian interventions are generally low-cost and well tolerated, their incorporation into multimodal treatment paradigms holds substantial promise for improving cerebrovascular resilience and long-term outcomes in the aging population [144] (Figure 7).

## 11. Precision Medicine and Biomarker-Guided Therapies in Aging-Related Cerebrovascular Disease

Aging-related cerebrovascular diseases exhibit marked biological heterogeneity, reflecting interindividual differences in genetic background, epigenetic regulation, immune competence, metabolic health, circadian integrity, and the burden of age-associated comorbid conditions such as hypertension, diabetes, and atrial fibrillation. This multilayered heterogeneity drives substantial variability in disease onset, progression, therapeutic responsiveness, and recovery trajectories among elderly patients, underscoring the inherent limitations of uniform, population-based treatment strategies [145,146]. Precision medicine approaches, guided by molecular, cellular, and systems-level biomarkers, aim to stratify patients according to dominant pathophysiological mechanisms and tailor interventions accordingly, representing a critical frontier in the management of aging-related cerebrovascular disease.

At the genetic level, genome-wide association studies [GWAS] have identified numerous loci associated with ischemic stroke subtypes, cerebral small vessel disease, and vascular cognitive impairment. These risk variants are distributed across pathways governing lipid metabolism, inflammatory signaling, endothelial function, extracellular matrix remodeling, and vascular integrity, highlighting the polygenic and mechanistically diverse nature of cerebrovascular disease [147,148]. Importantly, many GWAS-identified loci interact with aging-related biological processes, including immune senescence, mitochondrial dysfunction, and epigenetic drift, modulating disease susceptibility and clinical expression over the lifespan.

Genetic risk also influences therapeutic response and recovery potential. Variants affecting lipid handling, coagulation pathways, and inflammatory mediators may determine responsiveness to antithrombotic, anti-inflammatory, or metabolic interventions, while genetic determinants of vascular structure and extracellular matrix composition influence blood–brain barrier integrity and repair capacity [149,150]. These insights support the integration of genetic information into biomarker-guided frameworks aimed at optimizing preventive and therapeutic strategies in elderly populations. Importantly, genetic risk interacts strongly with aging-related processes, such as epigenetic drift and immune senescence, influencing disease susceptibility and therapeutic responsiveness [150]. Polygenic risk scores, while still evolving, may aid in identifying individuals at heightened cerebrovascular risk who could benefit from early preventive interventions.

Epigenetic biomarkers offer additional layers of precision by capturing dynamic, age-dependent regulatory changes that are not reflected at the DNA sequence level and that integrate genetic predisposition with environmental and lifestyle influences. Aging is associated with widespread alterations in DNA methylation patterns, histone modifications, and chromatin accessibility, collectively referred to as epigenetic drift. These changes exert profound effects on vascular gene expression, inflammatory signaling, mitochondrial function, and cellular stress responses, all of which are central to the pathophysiology of aging-related cerebrovascular disease [151,152].

In the cerebrovascular system, age-associated epigenetic remodeling influences endothelial identity, nitric oxide signaling, extracellular matrix regulation, and immune activation. Altered DNA methylation of genes involved in endothelial barrier function and angiogenesis has been linked to increased blood–brain barrier permeability and vascular stiffness, while histone modifications regulate transcriptional programs governing oxidative stress responses and inflammatory cascades [153,154]. In immune and glial cells, epigenetic reprogramming contributes to sustained pro-inflammatory phenotypes and impaired resolution of inflammation, amplifying neurovascular injury in the aging brain.

DNA methylation-based epigenetic clocks have emerged as particularly informative biomarkers of biological aging. Accelerated epigenetic aging, defined as a discrepancy between epigenetic and chronological age, has been associated with increased risk of stroke, cerebral small vessel disease, cognitive decline, and mortality, suggesting that epigenetic age may better reflect cerebrovascular vulnerability than chronological age alone [155,156]. These clocks capture cumulative exposure to metabolic stress, inflammation, and vascular injury, making them attractive tools for patient stratification and risk prediction. Importantly, epigenetic modifications are potentially reversible, positioning them as both biomarkers and therapeutic targets. Interventions that modulate metabolic pathways, inflammation, or circadian rhythms have been shown to influence epigenetic states, raising the possibility of biomarker-guided interventions aimed at decelerating biological aging and improving cerebrovascular outcomes [9,154,157]. Integrating epigenetic biomarkers into precision medicine frameworks may therefore enhance the ability to identify high-risk individuals, monitor therapeutic response, and tailor interventions in aging-related cerebrovascular disease. Epigenetic clocks, which estimate biological age based on DNA methylation patterns, have been associated with cerebrovascular risk and cognitive decline, suggesting potential utility in stratifying patients by biological rather than chronological age [151].

Circulating biomarkers derived from blood and cerebrospinal fluid provide minimally invasive and clinically accessible windows into cerebrovascular pathology, offering dynamic insights into disease severity, progression, and recovery potential. A broad spectrum of circulating inflammatory markers, endothelial-derived proteins, metabolites, and neuronal injury markers have been linked to stroke severity, blood–brain barrier [BBB] disruption, and long-term neurological and cognitive outcomes. Elevated levels of C-reactive protein, interleukin-6, vascular adhesion molecules, and markers of endothelial activation reflect systemic and cerebrovascular inflammation, while neurofilament light chain serves as a sensitive indicator of axonal damage and neurodegeneration in both acute and chronic cerebrovascular conditions [158,159].

Metabolomic biomarkers further capture age-related alterations in energy metabolism, oxidative stress, and lipid handling that influence cerebrovascular vulnerability and recovery. Changes in amino acid, lipid, and NAD^+^-related metabolic profiles have been associated with infarct size, BBB permeability, and post-stroke cognitive decline, underscoring the relevance of metabolic state as a determinant of outcome in elderly patients [160,161]. More recently, extracellular vesicles (EVs) have emerged as particularly informative biomarker platforms in aging-related cerebrovascular disease. EVs carry cell-type-specific molecular cargo, including microRNAs, long non-coding RNAs, proteins, and lipids, that reflect the functional and pathological state of their cells of origin. Endothelial-derived EVs report on vascular activation and BBB integrity, immune cell-derived EVs mirror inflammatory and senescent phenotypes, and neuron- or glia-derived EVs provide insights into neuronal injury, synaptic dysfunction, and neurodegeneration [92,94]. Importantly, EV-based biomarkers are particularly well suited to aging populations, as they integrate information across multiple pathological domains—including inflammation, metabolism, senescence, and neurovascular dysfunction—while allowing longitudinal monitoring of disease evolution and therapeutic response. Emerging studies suggest that EV cargo profiles can discriminate stroke subtypes, predict functional recovery, and identify patients most likely to benefit from targeted interventions, positioning EVs as powerful tools within precision medicine frameworks for aging-related cerebrovascular disease [162,163].

Aging-related alterations in EV profiles have been associated with chronic inflammation, BBB dysfunction, and impaired recovery, positioning EV-based biomarkers as powerful tools for patient stratification and therapy monitoring [164]. Multi-omics approaches integrating genomics, transcriptomics, epigenomics, proteomics, and metabolomics offer unprecedented opportunities to define molecular endotypes of aging-related cerebrovascular disease. By capturing multiple layers of biological regulation simultaneously, these integrative strategies enable comprehensive characterization of the complex interactions among genetic predisposition, age-associated regulatory changes, metabolic state, immune function, and environmental exposures that shape cerebrovascular vulnerability and recovery potential [165,166].

In aging-related cerebrovascular disease, multi-omics analyses have revealed convergent dysregulation of pathways related to inflammation, endothelial dysfunction, extracellular matrix remodeling, mitochondrial metabolism, and cellular senescence. Integration of transcriptomic and epigenomic data has elucidated age-dependent shifts in gene regulatory networks governing vascular integrity and immune activation, while proteomic and metabolomic profiling has identified circulating signatures associated with blood–brain barrier disruption, infarct severity, and long-term cognitive outcomes [167,168]. Importantly, multi-omics frameworks facilitate the identification of molecular endotypes—distinct mechanistic subgroups of patients characterized by dominant pathological drivers such as chronic inflammation, metabolic insufficiency, senescence burden, or impaired regenerative signaling. Defining these endotypes enables rational stratification of patients for targeted or combination therapies, increasing the likelihood of therapeutic efficacy while minimizing unnecessary exposure to ineffective treatments [169,170].

The clinical utility of multi-omics approaches is further enhanced by advances in computational biology, machine learning, and artificial intelligence, which enable integration of high-dimensional datasets and extraction of predictive patterns from longitudinal data. These tools support dynamic modeling of disease trajectories, prediction of treatment response, and real-time adaptation of therapeutic strategies—capabilities particularly valuable in aging populations with evolving pathophysiology and comorbidities [171,172]. Of note, longitudinal omics profiling enables tracking of disease evolution and treatment response over time, a critical consideration in chronic, age-associated conditions [165]. Precision medicine also extends to therapeutic timing and dosing, incorporating chronobiological principles and pharmacogenomic variability to optimize treatment efficacy in aging-related cerebrovascular disease. Age-related alterations in drug absorption, distribution, metabolism, and excretion—driven by changes in hepatic and renal function, blood–brain barrier permeability, and systemic inflammation—significantly influence pharmacokinetics and pharmacodynamics in elderly patients [173,174]. These factors contribute to substantial interindividual variability in therapeutic response and adverse event risk, necessitating individualized dosing strategies.

Chronobiological regulation further modulates drug efficacy and toxicity by influencing vascular tone, immune activity, and metabolic capacity across the circadian cycle. Disruption of circadian rhythms with aging alters temporal patterns of drug metabolism and target engagement, suggesting that time-of-day-dependent administration may enhance therapeutic benefit while reducing side effects. Chronotherapy has demonstrated efficacy in cardiovascular disease management and is increasingly recognized as relevant for cerebrovascular prevention and recovery [138,175].

Pharmacogenomic variability adds an additional layer of complexity, as genetic differences in drug-metabolizing enzymes, transporters, and molecular targets influence therapeutic response and bleeding or toxicity risk. Incorporating pharmacogenomic information may be particularly valuable in elderly patients receiving antithrombotic, anti-inflammatory, or metabolic therapies, where narrow therapeutic windows and polypharmacy are common [176,177]. Importantly, biomarker-guided selection of patients most likely to benefit from anti-inflammatory, senescence-targeted, metabolic, or regenerative therapies offers a powerful strategy to enhance translational success. Stratifying patients based on molecular signatures of inflammation, senescence burden, metabolic insufficiency, or impaired regenerative capacity can improve therapeutic targeting, reduce unnecessary exposure, and increase the probability of meaningful clinical benefit [170,172]. Together, integration of chronobiology, pharmacogenomics, and biomarker-guided stratification represents a critical step toward individualized, mechanism-based therapy for aging-related cerebrovascular disease.

Despite its considerable promise, implementation of precision medicine in aging-related cerebrovascular disease faces substantial scientific, logistical, and ethical challenges. A major limitation is the scarcity of large, well-characterized cohorts of elderly individuals with deep molecular phenotyping, longitudinal follow-up, and detailed clinical annotation. Aging-related heterogeneity, multimorbidity, and polypharmacy further complicate cohort design and data interpretation, underscoring the need for harmonized, age-inclusive study frameworks [178,179].

Standardization and validation of biomarker assays across platforms and centers represent additional hurdles. Variability in sample collection, processing, and analytical pipelines can limit reproducibility and impede clinical translation. These challenges are particularly pronounced for emerging biomarkers such as extracellular vesicles, multi-omics signatures, and epigenetic clocks, which require rigorous methodological consensus before widespread clinical adoption [180,181]. Furthermore, integration of high-dimensional datasets across molecular, imaging, and clinical domains demands advanced computational infrastructure and robust analytical frameworks capable of managing complexity without sacrificing interpretability.

Ethical and societal considerations are also central to the implementation of precision medicine in elderly populations. Issues related to data privacy, informed consent, equitable access to personalized therapies, and potential age-related bias in algorithmic decision-making must be carefully addressed. Older adults are often underrepresented in clinical research, raising concerns that precision medicine advances may inadvertently exacerbate health disparities if not inclusively designed [172,182].

Nonetheless, rapid advances in computational biology, artificial intelligence, and systems medicine are accelerating the feasibility of personalized approaches. Machine learning-based integration of multi-omics, imaging, and longitudinal clinical data enables identification of predictive patterns, dynamic disease modeling, and individualized therapeutic optimization. These tools are particularly well suited to addressing the complexity and temporal evolution of aging-related cerebrovascular disease, positioning precision medicine as an increasingly attainable goal rather than a distant aspiration [165,171].

**Figure 8:**
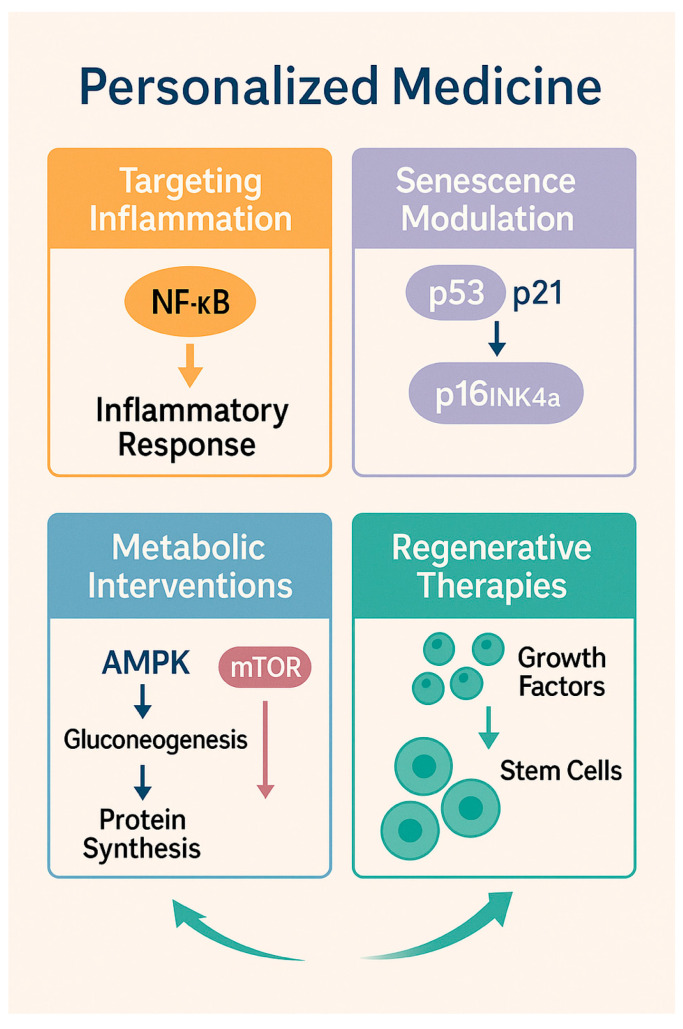
Molecular pathways underlying personalized therapeutic strategies in aging-related cerebrovascular disease. Single-panel schematic illustrating key molecular pathways targeted by personalized medicine approaches in aging-related cerebrovascular disease. Targeting inflammation focuses on suppression of chronic pro-inflammatory signaling pathways, particularly NF-κB- transcription, to reduce endothelial dysfunction and neuroinflammation. Senescence modulation aims to regulate cell cycle arrest and senescence-associated secretory phenotypes through pathways involving p53, p21, and p16^INK4a, thereby limiting senescence-driven vascular and neural damage. Metabolic interventions target energy-sensing and nutrient-signaling pathways, including AMPK activation and mTOR inhibition, to restore mitochondrial function, metabolic homeostasis, and cellular stress resilience. Regenerative therapies leverage stem cells and growth factor-mediated signaling to promote angiogenesis, neurovascular remodeling, and tissue repair. Integration of these pathway-specific interventions enables biomarker-guided, individualized therapeutic strategies tailored to the dominant molecular drivers of disease in each patient.

In summary, precision medicine and biomarker-guided therapies offer a transformative framework for addressing the complexity of aging-related cerebrovascular disease. By aligning therapeutic strategies with individual molecular and cellular profiles, these approaches hold the potential to improve prevention, enhance recovery, and ultimately reduce the burden of cerebrovascular disease in the aging population (Figure 8).

## 12. Future Directions and Translational Challenges

Despite rapid advances in understanding the molecular and cellular mechanisms underlying aging-related cerebrovascular diseases, translating these insights into effective clinical therapies remains a formidable challenge. Aging introduces unique biological, clinical, and methodological complexities that limit the direct applicability of many experimental findings. These include chronic low-grade inflammation, immune senescence, metabolic and mitochondrial decline, circadian dysregulation, and the accumulation of senescent cells, all of which profoundly modify disease mechanisms and therapeutic responsiveness [65,178]. Addressing these challenges will require a paradigm shift away from reductionist approaches toward age-aware, mechanism-driven, and integrative therapeutic strategies that explicitly account for the biology of aging.

One of the most critical obstacles to translation is the persistent reliance on young or middle-aged experimental models in preclinical cerebrovascular research. Such models fail to recapitulate essential features of the aged brain, including sustained neuroinflammation, immune dysfunction, impaired metabolic flexibility, altered neurovascular coupling, and senescence-associated remodeling of the neurovascular unit. Consequently, therapeutic approaches that demonstrate robust efficacy in young animals often show attenuated, delayed, or inconsistent benefits in aged systems [183,184]. These discrepancies highlight the limited predictive value of young-animal studies for clinical translation in elderly populations. Future research must therefore prioritize the systematic inclusion of aged animal models and, where feasible, models incorporating age-related comorbidities such as hypertension, diabetes, dyslipidemia, and atrial fibrillation to better reflect the clinical reality of cerebrovascular disease in older adults [185].

Another major challenge lies in the temporal complexity of aging-related cerebrovascular disease. Aging alters not only baseline physiological states but also the timing, magnitude, and resolution of injury responses and repair mechanisms. Inflammatory activation, metabolic failure, blood–brain barrier disruption, and regenerative processes exhibit age-dependent kinetics, suggesting that therapeutic windows for anti-inflammatory, metabolic, regenerative, and senescence-targeted interventions may differ substantially between younger and older individuals [11,121]. Determining optimal timing, dosing, and sequencing of therapies—particularly across acute, subacute, and chronic phases of cerebrovascular injury—will be essential for successful translation.

Chronobiology-informed approaches further add complexity to this temporal landscape by introducing time-of-day-dependent variability in vascular function, immune responsiveness, and drug metabolism. While circadian disruption in aging complicates therapeutic planning, it also offers opportunities to refine therapeutic precision through chronotherapy and time-aligned interventions [145,175]. Integrating temporal biology into experimental design and clinical trial frameworks may therefore enhance both efficacy and safety of emerging therapies in elderly patients.

Safety considerations are particularly salient in elderly populations, where aging is accompanied by reduced physiological reserve, multimorbidity, polypharmacy, and heightened vulnerability to adverse drug reactions. Age-related changes in hepatic and renal clearance, blood–brain barrier permeability, immune responsiveness, and mitochondrial function significantly alter drug pharmacokinetics and pharmacodynamics, increasing the risk of toxicity even at standard doses [173,186]. These factors necessitate careful dose optimization and vigilant monitoring when introducing novel therapies in older patients.

Interventions targeting fundamental aging mechanisms, such as senolytics, immune modulators, and metabolic reprogramming agents, pose particular safety challenges due to their broad biological effects. Senolytic therapies, while promising, may disrupt tissue homeostasis, impair wound healing, or compromise immune surveillance if senescent cell clearance is excessive or poorly timed [80,187]. Similarly, immune-modulatory strategies risk tipping the balance toward immunosuppression or uncontrolled inflammation in elderly individuals with pre-existing immune dysfunction.

Metabolic reprogramming agents, including mTOR inhibitors and NAD^+^-boosting compounds, also require cautious evaluation in aged populations. While these interventions can enhance cellular stress resistance and vascular function, long-term effects on immune competence, glucose metabolism, and organ function remain incompletely characterized, particularly in the context of polypharmacy and chronic disease [82,118]. Drug–drug interactions are of special concern, as elderly patients frequently receive anticoagulants, antihypertensives, antidiabetic agents, and lipid-lowering therapies that may interact with emerging treatments. Accordingly, rigorous assessment of benefit–risk ratios in aged and comorbid cohorts will be indispensable before widespread clinical adoption of aging-targeted cerebrovascular therapies. Age-specific dosing strategies, adaptive trial designs, and long-term safety monitoring must be integrated into translational pipelines to ensure that therapeutic advances translate into meaningful and safe clinical benefit for elderly patients [178,188].

The implementation of precision medicine in aging-related cerebrovascular disease presents both promise and complexity. While multi-omics profiling, advanced imaging, and circulating biomarkers offer unprecedented insights into individual disease mechanisms, integrating these data into clinically actionable frameworks remains challenging. Standardization of biomarker assays, validation across diverse populations, and development of robust computational tools for data integration are critical next steps. Moreover, ethical considerations surrounding data privacy, access to personalized therapies, and equitable inclusion of older adults in research must be carefully addressed.

From a clinical trial perspective, innovative study designs will be required to accommodate the heterogeneity of aging populations. Adaptive trials, platform trials, and biomarker-enriched enrollment strategies may improve efficiency and translational relevance. Importantly, meaningful clinical endpoints in elderly patients may extend beyond traditional measures of survival or infarct size to include functional independence, cognitive outcomes, and quality of life. Aligning therapeutic goals with patient-centered outcomes will be essential for clinical impact.

Finally, future therapeutic success in aging-related cerebrovascular disease will likely depend on combinatorial and multimodal approaches that address the inherent biological complexity of the aging brain. Rather than arising from a single dominant pathway, aging-related cerebrovascular disease reflects the convergence of chronic inflammation, metabolic and mitochondrial dysfunction, vascular instability, cellular senescence, impaired circadian regulation, and diminished regenerative capacity. These interconnected processes interact dynamically across the neurovascular unit and evolve over time, rendering monotherapeutic strategies insufficient to achieve durable clinical benefit [2,147].

Importantly, combinatorial strategies must be guided by mechanism-based stratification and temporal precision. The optimal composition, sequencing, and timing of interventions are likely to vary across disease stages and patient subgroups, necessitating integration of biomarkers, chronobiological insights, and longitudinal monitoring to dynamically adapt therapeutic regimens [170,172]. In this context, precision medicine frameworks provide the essential infrastructure for implementing multimodal therapies safely and effectively in elderly populations. Targeting a single pathogenic pathway in isolation is therefore unlikely to produce durable therapeutic benefit in aging-related cerebrovascular disease. The multifactorial nature of cerebrovascular aging—driven by intersecting inflammatory, metabolic, vascular, senescent, and regenerative disturbances—necessitates rational combination therapies that simultaneously modulate complementary biological processes. Biomarker-guided strategies that tailor therapeutic combinations to individual molecular and cellular profiles offer a promising approach to overcoming interindividual heterogeneity and improving therapeutic responsiveness [147,170].

Integrating pharmacological interventions with biological therapies, lifestyle modification, and chronobiological alignment may further enhance efficacy by reinforcing endogenous repair mechanisms and minimizing adverse effects. Lifestyle-based interventions targeting physical activity, nutrition, sleep, and circadian regularity influence many of the same pathways engaged by pharmacological agents, including inflammation, metabolism, mitochondrial function, and vascular health, and may act synergistically when incorporated into multimodal treatment paradigms [106,107,108,109,110,111,112,113,114,115,116,117,118,119,120,121,122,123,124,125,126,127,128,129,130,131,132,133,134,135,136,137,138,139,140,141,142,143,144,145,189,190]. Together, these integrative, biomarker-informed strategies hold substantial promise for improving neurovascular resilience, functional recovery, and long-term outcomes in the growing aging population affected by cerebrovascular disease.

## 13. Conclusions

Aging fundamentally reshapes the biological landscape of the cerebrovascular system, transforming vascular structure, immune responses, metabolic resilience, and regenerative capacity. As a result, aging-related cerebrovascular diseases arise from the convergence of endothelial dysfunction, chronic neuroinflammation, cellular senescence, metabolic and mitochondrial decline, circadian dysregulation, and impaired intercellular communication within the neurovascular unit. Traditional therapeutic approaches, largely developed in younger populations, fail to adequately address these age-specific mechanisms, underscoring the urgent need for new, biologically informed strategies.

This narrative review highlights emerging therapeutic avenues that directly target the molecular hallmarks of cerebrovascular aging. Advances in immunomodulation, senescence-targeted interventions, metabolic and mitochondrial therapies, stem cell-based and extracellular vesicle-mediated regeneration, RNA-based approaches, and chronobiology-informed treatments collectively represent a paradigm shift from symptom-oriented care toward mechanism-driven intervention. Importantly, these strategies emphasize restoration of neurovascular integrity, resolution of maladaptive inflammation, and enhancement of endogenous repair processes rather than isolated neuroprotection. Precision medicine frameworks further expand the therapeutic horizon by integrating genetic, epigenetic, metabolic, and circulating biomarkers to stratify patients and guide individualized treatment selection. Such approaches acknowledge the heterogeneity of aging-related cerebrovascular disease and offer the potential to optimize efficacy while minimizing risk in elderly populations. However, successful clinical translation will require rigorous validation in age-appropriate models, careful attention to safety and timing, and innovative clinical trial designs tailored to the complexity of aging.

## Data Availability

No new data were created or analyzed in this study. Data sharing is not applicable to this article.
